# Risk perceptions and adaptive strategies in sociobiodiversity value chains: a case study with pink pepper tree (*Schinus terebinthifolia* Raddi) extractivists in Northeastern Brazil

**DOI:** 10.1186/s13002-026-00869-6

**Published:** 2026-03-27

**Authors:** Ramon Salgueiro Cruz, Italo Jose Silva Amaral, Iasmim Silva Oliveira, Guillherme Ramos Demetrio, Patrícia Muniz de Medeiros, Rafael Ricardo Vasconcelos da Silva

**Affiliations:** 1 Laboratory of Biocultural Ecology, Conservation and Evolution (LECEB), Campus of Engineering and Agricultural Sciences (CECA), Federal University of Alagoas, Rio Largo, AL, 57100-000 Brazil; 2 Laboratory of Plant Ecology (LEVE), Federal University of Alagoas, Arapiraca Campus, Penedo Educational Unit, Penedo, 57200-000 Alagoas Brazil

**Keywords:** Sociobiodiversity, Extractivism, Climate change

## Abstract

Environmental changes are at the core of scientific discussions and represent imminent risks to numerous productive activities, including the value chains of sociobiodiversity products. In the face of these risks, local populations develop perceptions and adaptive strategies shaped by their sociocultural contexts. In this study, we sought to identify the factors that explain risk perceptions of environmental changes and adaptive strategies within the value chain of the Brazilian pepper tree (*Schinus terebinthifolia* Raddi.), whose fruits are harvested through extractivism and marketed as pink pepper, in the São Francisco River mouth region, northeastern Brazil. We tested the following hypotheses: H1. Female extractivists, younger individuals, those with higher income, higher education, longer residence, and/or access to more information sources perceive a greater number of risks and implement a higher number of adaptive strategies. H2. Female extractivists, younger individuals, those with higher income, higher education, longer residence, and/or access to more information sources assign greater severity to the most frequently occurring risks. H3. Extractivists with higher participation in associations, prior experience with risks, longer extractivism experience, ownership of collection areas, unrestricted access to collection areas, higher profitability, and/or higher productivity perceive a greater number of risks and adopt more adaptive strategies. H4. Extractivists with higher participation in associations, prior experience with risks, longer extractivism experience, ownership of collection areas, unrestricted access to collection areas, higher profitability, and/or higher productivity assign greater severity to the most frequently occurring risks. Data collection involved a participatory workshop followed by semi-structured interviews with 53 extractivists from 14 different communities in the region. The most frequently cited risk was “excessive rainfall.” We found that younger and more experienced extractivists assigned greater severity to environmental risks, supporting hypotheses 2 and 4. The scarcity of specific strategies to cope with extreme climatic events highlights the urgent need for integrated public policies capable of articulating traditional knowledge, climate justice, and the valorization of extractivist practices.

## Highlights

- We evaluated how socioeconomic, environmental, and experiential factors shape risk perceptions among pink pepper extractivists.
- Age and extractive experience were key predictors of perceived severity toward environmental risks.
- Limited institutional support and socioeconomic constraints reduce adaptive capacity in traditional communities.
- Our findings provide relevant insights for designing climate-resilient strategies and strengthening biocultural conservation policies.

## Introduction

Environmental changes play a prominent role in contemporary public debates and global concerns, as they pose risks to human well-being, productive activities, and the sustainability of socioecological systems [1, 2]. However, perceptions of these risks vary among individuals and social groups [3–6]. On one hand, scientific evidence and predictions about these changes are increasingly evident to human populations; on the other hand, certain sectors deny these findings [7, 8]. In this context, studies on risk perception have contributed to assessing the vulnerability of social groups’ livelihoods and to developing strategies to mitigate the impacts arising from such risks [9–11].

Environmental change risks can be understood as the likelihood of adverse effects resulting from natural or anthropogenic phenomena [12, 13], including droughts, floods, fires, and deforestation. Thus, the social perceptions of these risks serve as indicators of environmental sensitivity and can be used to anticipate the adoption of adaptive strategies aimed at avoiding, mitigating, or coping with their potential impacts [14–16]. However, many factors may shape how individuals and social groups perceive risks and adopt adaptive strategies in response to changes in their environments [17–19], with socioeconomic and productive factors standing out as particularly influential [20, 21].

Within the context of the present study, we highlight the Sociobiodiversity Value Chains (SVCs) as productive systems that are particularly sensitive to environmental changes, as they integrate ecological, economic, and sociocultural dimensions in the generation of goods and services associated with the sustainable use of biodiversity. These chains involve several stages—from the productive base, generally structured around the extractivism of non-timber forest products, through processing and distribution, to final consumption. Institutional, organizational, and non-monetary value dimensions are also integrated throughout the entire production process [22–24].

Although studies on value chains have advanced in identifying socioeconomic, ecological, technological, and organizational weaknesses across different chain segments [25], the effects of environmental changes on these chains remain poorly understood. This knowledge gap becomes even more evident when we focus on the risk perceptions of extractivist actors involved in the productive base of these value chains, as well as their perceptions of risk severity and adaptive strategies in response to environmental changes.

Bridging this gap becomes increasingly important in the face of the growing intensification of extreme climatic events and socioenvironmental uncertainties, which directly affect extractivist activities involving sociobiodiversity products. Understanding how socioeconomic and productive factors shape perceptions of risk, severity, and adaptive strategies to environmental changes has direct implications for the sustainability and governance of these chains. Such information can inform public policies, management actions, and territorial development initiatives that strengthen the socioecological resilience of SVCs and promote biocultural conservation strategies through the sustainable use of plant resources.

### Factors modulating risk Perceptions, Severity, and adaptive strategies

In this study, we investigated which factors most strongly influence risk perceptions, the perceived severity of risks, and adaptive strategies in response to environmental change risks within extractivist-based sociobiodiversity value chains. Accumulated evidence suggests that socioeconomic and productive characteristics modulate how different individuals and social groups perceive risks and develop adaptive strategies [17–19].

Regarding the understanding of the severity attributed to perceived risks, the concept of “objective risk” is particularly relevant [5, 12]. This perspective acknowledges that recurrent exposure to more frequent risks tends to calibrate perception, making it more aligned with empirically observed severity. Therefore, the assessment of factors modulating the severity attributed to a risk must initially take into account the incidence of that risk, since the frequency and intensity of exposure represent important aspects in the social construction of perceived severity.

### Socioeconomic factors

Among the most studied socioeconomic factors is gender, with evidence suggesting that women, particularly in rural communities, tend to perceive environmental risks more acutely, which is associated with their direct involvement in care and subsistence activities [14, 26, 27]. Age, however, shows more complex relationships with risk perceptions. Recent large-scale studies indicate that younger individuals perceive higher risks from climate change [28, 29], while other studies report no significant influence of age [30, 31].

Education and income are also associated with risk perception, as they influence access to and understanding of information about environmental issues. Higher levels of education and income tend to enhance the capacity to identify risks and adopt strategies to address them [32, 33]. Similarly, access to information—whether technical, institutional, or through media—also shapes risk perception, with more diverse and reliable sources increasing individuals’ sensitivity to environmental changes and their capacity to implement adaptive strategies [7, 8, 32].

Length of residence in an area and occupation may also exert significant influence. Residents who have lived longer in a territory tend to accumulate greater local ecological knowledge but may normalize certain environmental changes, thereby reducing perceived risk or its severity [34–36]. Conversely, individuals whose occupations are directly tied to the natural environment—such as extractivists, fishers, or traditional farmers—tend to perceive environmental change risks more strongly, given their direct dependence on natural resources for subsistence [37].

Based on these considerations, we tested the following hypothesis:

H1. Female extractivists, younger individuals, those with higher income, higher education, longer residence, and/or access to more information sources perceive a greater number of risks and implement a higher number of adaptive strategies.

H2. Female extractivists, younger individuals, those with higher income, higher education, longer residence, and/or access to more information sources assign greater severity to the most frequently occurring risks.

### Productive factors

Productive aspects also play a decisive role in shaping risk perceptions and the development of adaptive strategies. Experience in the productive activity, for example, can lead to more realistic perceptions of environmental change risks [34]. Individuals with longer experience in specific value chains possess a repertoire that allows them to better recognize the effects of environmental changes within that chain. Conversely, a lack of prior experience with risks tends to limit the ability to anticipate threats and develop adaptive strategies [5, 8, 12].

Collective engagement in the production process, such as participation in associations, can strengthen access to information and the development of collective strategies to face risks [17, 38, 39]. However, structural and institutional factors, such as lack of land tenure and guarantees of access to harvesting areas, create an environment of uncertainty that reduces investment and engagement in sustainable management and conservation actions. Situations of land insecurity or restricted access tend to diminish extractivists’ investment in adaptive strategies [40, 41].

Finally, the profitability and productivity of the activity also influence risk perception in value chains, as the negative impacts of environmental changes can more tangibly affect the income and livelihoods of those engaged in more profitable and productive value chain activities [21, 42].

Based on these considerations, we postulate the following hypotheses:

H3. Extractivists with higher participation in associations, prior experience with risks, longer extractivism experience, ownership of collection areas, unrestricted access to collection areas, higher profitability, and/or higher productivity perceive a greater number of risks and adopt more adaptive strategies.

H4. Extractivists with higher participation in associations, prior experience with risks, longer extractivism experience, ownership of collection areas, unrestricted access to collection areas, higher profitability, and/or higher productivity assign greater severity to the most frequently occurring risks.

## Materials and methods

### Study area

The study was conducted in the municipality of Piaçabuçu, located in the southern region of Alagoas state, Brazil. The municipality is bordered to the north by Penedo and Feliz Deserto, to the south by the São Francisco River, to the east by the Atlantic Ocean, and to the west again by Penedo [43]. Piaçabuçu is situated approximately 143 km from the state capital, Maceió, and has a territorial area of 241.96 km² [44], with an estimated population of 15,908 inhabitants [45]. The regional climate is classified as humid tropical with a dry summer and a rainy season concentrated in autumn–winter, with an average annual precipitation of 1217.5 mm and a mean annual air temperature of 24.9 °C. However, the region is characterized by marked interannual precipitation variability, with occasional periods of excessive rainfall that can directly affect extractivist activities. Annual precipitation totals recorded in 2022 and 2023 substantially exceeded the historical regional average, ranging from approximately 19.4% to 44.5% above the mean in 2022 and 11% to 41% in 2023, according to variations observed in official data provided by the National Water and Sanitation Agency (ANA). These results indicate that both years can be classified as atypically wet within the analyzed historical series, which comprised records available between 2011 and July 2025.

**Fig. 1:**
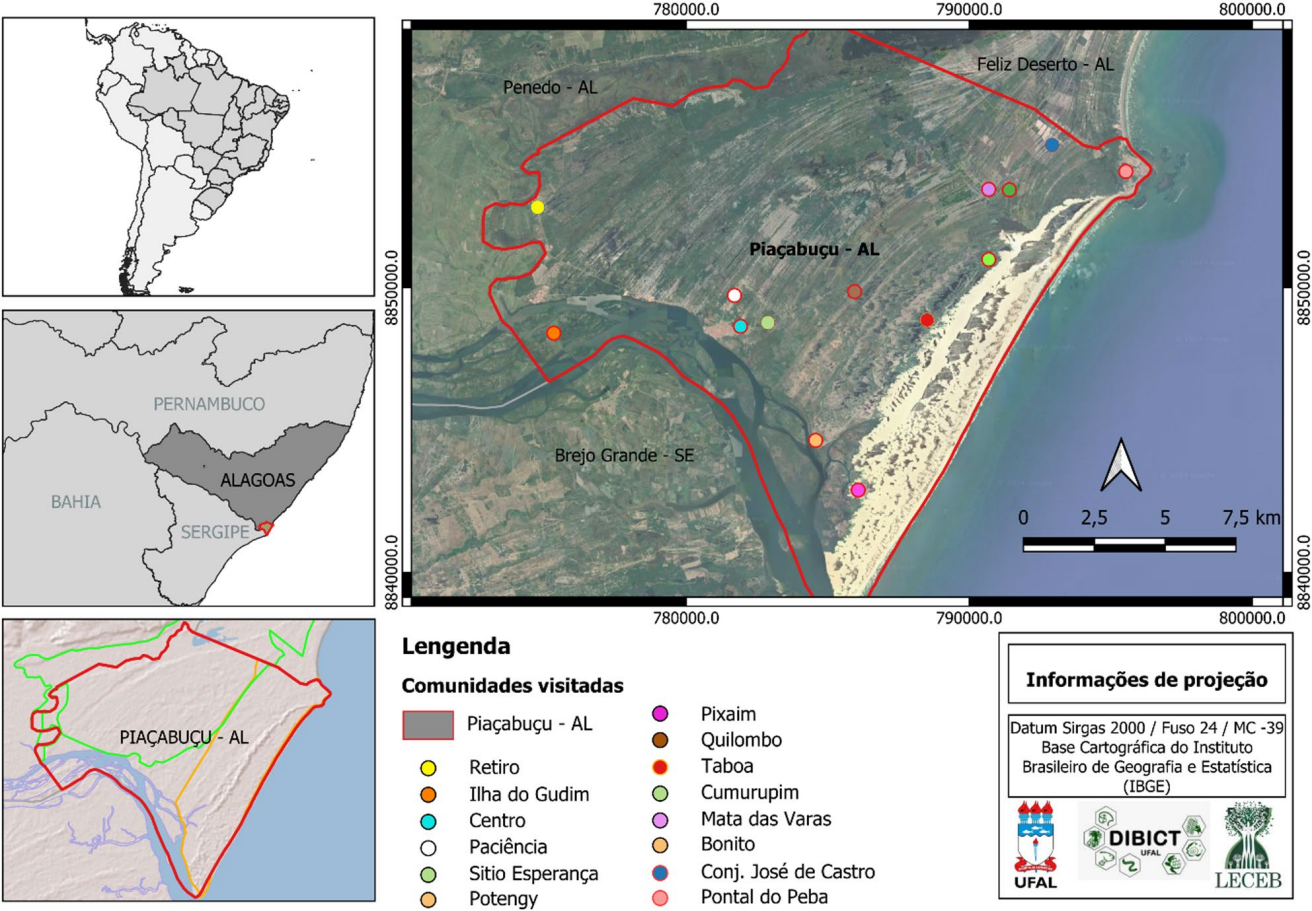
Location of the municipality of Piaçabuçu, Alagoas, Brazil, highlighting the communities visited

The municipality contains two sustainable-use conservation units, classified as Environmental Protection Areas (APAs). The first is the Piaçabuçu APA, covering 9,107.01 hectares and managed by the federal agency Instituto Chico Mendes de Conservação da Biodiversidade (ICMBio). The second is the Marituba do Peixe APA, encompassing 18,600 hectares and managed by the state agency Instituto do Meio Ambiente do Estado de Alagoas (IMA). The latter includes portions of the municipalities of Piaçabuçu (10,867.31 ha), Feliz Deserto (3,937.88 ha), and Penedo (3,804.18 ha). Overall, approximately 82% of the territory of Piaçabuçu falls within APA zones (Fig. 1). Given this extensive territorial overlap with protected areas, most extractivist communities involved in this study are located within Conservation Units. Due to zoning regulations, some communities fall within more restrictive zones, which may influence local resource-use practices and interactions with external actors. This regulatory context was occasionally associated with reluctance to participate in the research or with initial denials of extractivist activity.

The economy of Piaçabuçu is based on commerce, services, and agriculture. In the primary sector, coconut cultivation and extractivism are particularly notable. The municipality also hosts the largest shrimp farm in the northeastern region of Brazil. Tourism plays a significant role in the local economy, with emphasis on the mouth of the São Francisco River.

**Fig. 2:**
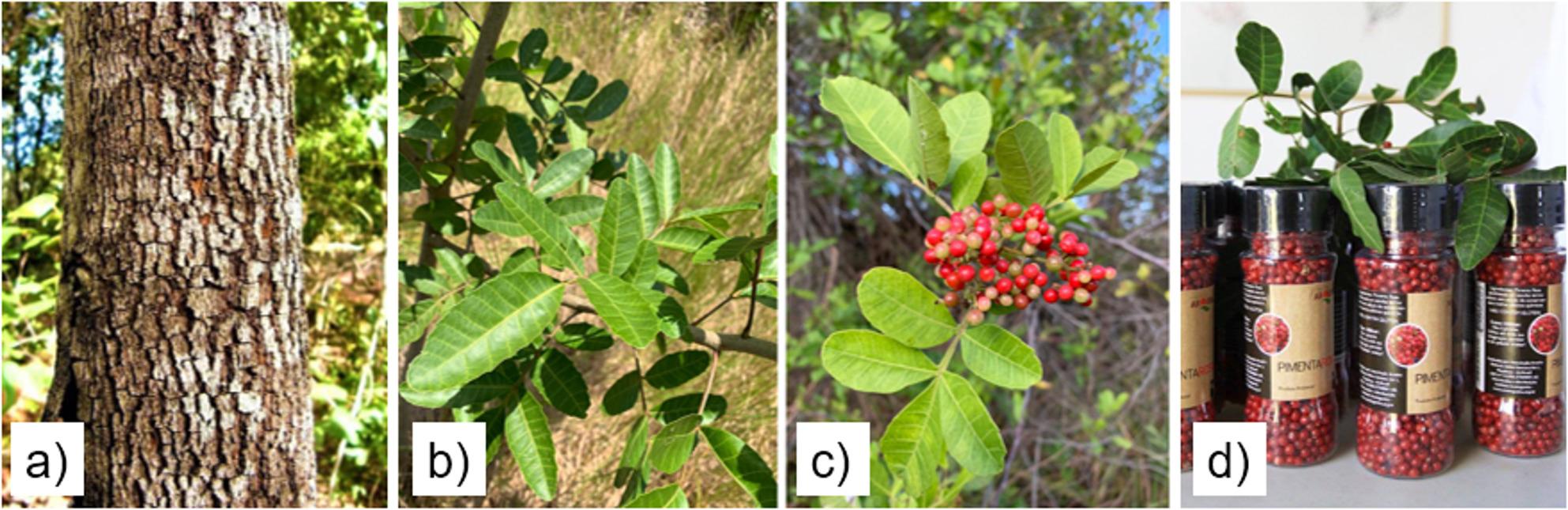
Aroeira (Schinus terebinthifolia Raddi); (**a**) Stem; (**b**) Leaves; (**c**) Fruit; (**d**) Marketed products

Data collection focused on extractivists of Brazilian pepper tree (*Schinus terebinthifolia* Raddi) (Fig. 2), initially organized under the Associação Aroeira, founded in 2011 in Piaçabuçu, AL. The association brought together over 50 native fruit extractivists from different local communities. With the growth of activities and increased group representation, the organization evolved into the Cooperativa Aroeira, expanding its scope and ensuring greater formalization of extractivism in the region. The fruits of the Brazilian pepper tree are marketed as pink pepper, forming one of the best-structured sociobiodiversity value chains in the region.

## Legal aspects

The project was submitted to the Research Ethics Committee of the Federal University of Alagoas, in compliance with the requirements of Resolution No. 466/2012 of the National Health Council (CAAE 58122122.4.0000.5013) and the Biodiversity Authorization and Information System – SISBIO (94419). Extractivists who agreed to participate in the study signed a Free and Informed Consent Form (FICF), declaring that they were aware of the research objectives and methods and authorizing the use of the information they provided.

### Data collection

#### Preliminary stage

*Participatory workshop to develop the guides for semi-structured interviews.*

Initially, a Participatory Rapid Appraisal (PRA) workshop [46] was conducted with the participation of 13 extractivists who were members of the Associação Aroeira. The objective was to generate preliminary information based on the following question: (1) What are the problems that most threaten the activities involving the collection and sale of Brazilian pepper tree fruits?

During the workshop, the tool called “Criteria and Options Matrix” [46, 47] was applied, through which participants were encouraged to list the main perceived risks related to the collection and sale of Brazilian pepper tree fruits. After listing the risks identified by participants, they were asked to assign scores on a Likert scale from one to five for the severity and frequency they attributed to each perceived risk (1 = very low, 2 = low, 3 = medium, 4 = high, 5 = very high). Finally, participants were asked to list all corresponding adaptive strategies for each of the identified risks (Table 1).

**Table 1:** Perceived risks related to sociobiodiversity value chains and adaptive strategies indicated by extractivists in a participatory workshop. Associação Aroeira, Piaçabuçu, Brazil

Risk categories	Risks	Comments	G	F	Strategies
Environmental Changes	Increased Distance or Difficulty of Access	*It increases transportation challenges and makes collection more difficult.*	2	4	* Change the mode of transportation * Adjust the schedule (go earlier)
	Excessive Rainfall	*Fruit rot on the tree before ripening*	4	3	No strategies are in place.
	Temperature Increase (excessive sun)	*Decrease and difficulty in harvesting*,* leading to fruit rot before collection.*	2	3	No strategies are in place.
	Deforestation	*Removal of trees and harvesting areas for stakes*,* fences*,* and monoculture plantations (sugarcane).*	5	4	Raise awareness among landowners Reforestation Change the harvesting area
	Fire/Wildfire	*People who burn down trees completely*,* including the roots*,* to prevent anyone from harvesting.*	1	3	Raise awareness among landowners
Accidents	Natural Accidents	*Risk of bites or stings from snakes*,* wasps*,* and bees*,* as well as contact with urticant plants.*	4	4	Scare the animal away Keep a safe distance from the animal or urticant plant
	Livestock/Domestic Animals	*Cows around the harvesting areas chase people and hinder the work.*	2	4	Keep a safe distance from the animal.
Human Activities	Violence Against Women	*Risk of being attacked by men near the harvesting areas.*	5	5	Harvest in groups Avoid certain locations
	Market Value Decline	*Risk of a decrease in product price.*	5	-	-

Where G = severity score and F = frequency score of the risk

During the workshop, participants identified eight perceived risks in the pink pepper value chain. Five of these were related to environmental changes, two to accidents, and one to deliberate human actions. In the discussions, “market value decline” was unanimously considered a very high-severity risk (score 5), perceived as an external factor beyond environmental or harvesting conditions, linked to commercial dynamics over which the extractivists have little or no control. For this reason, participants did not assign frequency scores or identify adaptive strategies associated with this risk. Nevertheless, its mention highlights the importance of economic dimensions in extractivism vulnerability. Despite this, the risk of “market value decline” was considered in the subsequent data collection stage through semi-structured interviews. However, its empirical occurrence was low, with only two informants recognizing this risk and reporting severity and frequency scores, as well as indicating adaptive strategies. It is important to note that only the information obtained from the interviews composed the quantitative database used in the statistical analyses for hypothesis testing.

In addition, participants reported a set of adaptive strategies to address the listed risks (Table 1). The information and discussions generated during the workshop allowed us to understand local expressions, subjectivities, and interpretations of extractivists regarding risks and adaptive strategies in the pink pepper value chain. This knowledge enabled the development of a semi-structured interview guide that was more closely grounded in local realities and challenges.

## Semistructured interviews

To conduct the interviews, participants were selected by non-probabilistic sampling, using the snowball technique [48], whereby at the end of each interview, the interviewee indicated other people who worked in the pink pepper value chain and their respective communities. This process was repeated until a “saturation point” was reached, i.e., the moment when no new names emerged [49]. Initially, extractivists of aroeira (*S. terebinthifolia*) who were members of the Aroeira Association were selected for interviews. Next, the extractivists who were referred and who were not members of the Association were interviewed. To participate in the research, one of the prerequisites for interviewees was to have current or previous experience with aroeira extractivism. Thus, 53 people agreed to participate in the research, residing in 14 different communities in the municipality of Piaçabuçu-AL (Fig. 1). A small number of individuals declined participation, mainly due to temporary unavailability during field visits, and whenever possible, interviews were rescheduled. In a few cases, initial reluctance was also associated with the regulatory context of protected areas and zoning restrictions.

Initially, forms were used to characterize the socioeconomic profile, recording the following information: age, gender, education, length of residence in the region, family income, sources of income, participation in cooperatives and/or associations. Also at this stage, respondents were asked about their main sources of information, with the following options: acquaintances/family members, newspapers/magazines, radio, television, and the internet. For analysis purposes, each selected source of information was equivalent to 1 point, with a total of 0 to 5 points, so that higher scores would indicate greater access to information.

Next, questions were asked about the participants’ productive profile, including: length of experience with the species/value chain, previous experience with risks (yes/no), marketing of other socio-biodiversity products (yes/no), and access to Technical Assistance and Rural Extension (TAR) services related to the species/value chain.

Subsequently, participants were asked to answer questions related to their perceptions of environmental change risks and adaptation strategies to these changes in value chains, including: a free list of perceived risks in the species value chain, assigning scores on a Likert scale from one to five, for the severity and frequency they attributed to each perceived risk (where 1 = very low, 2 = low, 3 = medium, 4 = high, and 5 = very high). Finally, they were asked to indicate adaptive strategies for each risk.

### Data analysis

Descriptive frequency analyses (relative frequencies) were performed to characterize the socioeconomic and productive profiles of the extractivists interviewed (Tables 5 and 6). In addition, the risk incidence index [9, 11, 50] was calculated, which represents the proportion of individuals who mentioned a particular risk, calculated by the equation Ij = nr/nj, where nr represents the number of mentions of the risk, and nj represents the total number of informants, such that the results can vary from 0 (no mentions) to 1 (mentioned by all informants). We also calculated the average severity and frequency scores assigned to the risks perceived by the extractivists. The risks with the highest incidence and highest average scores in terms of severity and frequency were considered the most relevant for participants in the pink pepper value chain.

To test Hypotheses 1 and 3 (Table 2), the Generalized Linear Models (GLM) approach with Poisson distribution was applied, which is suitable for analyzing the influence of socioeconomic and productive attributes on the number of perceived risks and adaptive strategies adopted. The choice of this model was justified by the nature of the dependent variables—the number of perceived risks (Nr) and the number of adaptive strategies (Nest)—both characterized as count variables, for which Poisson distribution is recommended.

Initially, a Poisson model was adjusted for each dependent variable, considering separately the independent variables of socioeconomic and productive nature as predictors (Table 2).

The selection of variables was conducted using the step() function, using the Akaike Information Criterion (AIC) as a reference for model optimization. This iterative procedure allowed the model to be refined in stages, discarding variables that did not contribute significantly to the adjustment, with the aim of achieving a simpler model with a better fit. The selection of variables took into account the relevance of each one in predicting the number of perceived risks (Nr) and the number of adaptive strategies (Nest).

To test Hypotheses 2 and 4 (Table 2), the Cumulative Link Model (CLM) was applied to investigate how socioeconomic and productive attributes influence the severity attributed to the risk of higher incidence. The choice of this model was based on the ordinal nature of the dependent variable—the severity scores assigned to risk—measured using a Likert scale. This type of model is suitable for ordinal variables, as it allows modeling the probability of an individual assigning a higher or lower severity score, considering the effects of the predictor variables included in the analysis.

Preliminarily, the CLM model was adjusted with the inclusion of all explanatory variables related to socioeconomic and productive profiles (Table 2).

To improve the model, the step() function was used, based on the Akaike Information Criterion (AIC), to conduct automated variable selection. This iterative procedure eliminated variables that did not contribute significantly to the adjustment, aiming to obtain a more parsimonious model without compromising its suitability for the data. The final choice of variables considered their relevance in explaining the severity attributed to the risk of higher incidence.

Once the selection stage was complete, the final model incorporated the variables length of experience in extractivism (tex) and ownership regime of the collection area (reg). The influence of these factors on the severity attributed to risk was assessed using the coefficients estimated in the CLM model, which express the direction and magnitude of the associations.

All statistical analyses were conducted in the RStudio environment (version 2024.12.0 + 467). The generalized linear models (GLM), used for count data with Poisson distribution, were adjusted with functions from the R base package. The cumulative link models (CLM), applied to ordinal variables, were adjusted with the ordinal package specific to this type of analysis [51].

Considering the sample size (*N* = 53), we acknowledge that the ratio between observations and initial predictors may represent a limitation for regression-based analyses. To reduce the risk of overfitting, automated variable selection using the step() function based on the Akaike Information Criterion (AIC) was applied, resulting in parsimonious final models composed of a limited number of predictors. Therefore, model interpretations were conducted with caution, emphasizing effect direction and consistency rather than statistical significance alone.

**Table 2:** Dependent and independent variables used in testing each hypothesis

Hypothesis	Dependent variables	Independent variables
H1. Female extractivists, younger individuals, those with higher income, higher education, longer residence, and/or access to more information sources perceive a greater number of risks and implement a higher number of adaptive strategies.	Number of perceived risks (Nr); Number of adaptive strategies (Nest)	Socioeconomic profile: gender (gen), length of residence (tem), age, occupation in nature (ocn), extractive occupation (oce), education (esc), monthly income (ren), sources of information (fon)
H2. Female extractivists, younger individuals, those with higher income, higher education, longer residence, and/or access to more information sources assign greater severity to the most frequently occurring risks.	Severity attributed to the risk of highest incidence	
H3. Extractivists with higher participation in associations, prior experience with risks, longer extractivism experience, ownership of collection areas, unrestricted access to collection areas, higher profitability, and/or higher productivity perceive a greater number of risks and adopt more adaptive strategies.	Number of perceived risks (Nr); Number of adaptive strategies (Nest)	Production profile: Participation in association (ass), level of participation (nas), previous experiences with risk (exp), length of experience in extractivism (tex), ownership regime of the collection area (reg), accessibility of collection areas (ace), profitability (ren), productivity (pro)
H4. Extractivists with higher participation in associations, prior experience with risks, longer extractivism experience, ownership of collection areas, unrestricted access to collection areas, higher profitability, and/or higher productivity assign greater severity to the most frequently occurring risks.	Severity attributed to the risk of highest incidence	

## Results and discussion

### Characterization of the socioeconomic and productive profiles of Pink pepper extractors

Pink pepper extractors were predominantly women (83.00%), one third aged over 61. More than half of the extractors (50.94%) had low levels of education (incomplete elementary school). The average monthly income reported by 64.15% of participants was up to one minimum wage (1,518.00 Brazilian reais or US$266.78 at the time of the survey) (Table 3).

**Table 3:** Characterization of the socioeconomic profile of Pink pepper extractors. Piaçabuçu, Alagoas, Brazil

Socioeconomic indicator	Percentage of respondents
**Gender**	
Women	83.00%
Men	17.00%
**Age group**	
18 to 30	9.43%
31 to 40	18.87%
41 to 50	18.87%
51 to 60	22.64%
61 or older	30.18%
**Education**	
No formal education	18.87%
Incomplete elementary school	50.94%
Incomplete high school	3.73%
Complete high school	24.52%
Complete higher education	1.86%
**Monthly income**	
Up to 1 salary	45.28%
From 1 to 2 salaries	39.62%
From 2 to 3 salaries	7.54%
From 3 to 5 salaries	5.66%
Above 5 salaries	1.86%
**Access to information**	
1 source of information	51.94%
2 sources of information	34.88%
3 sources of information	11.32%
4 sources of information	1.86%

In terms of their productive profile, many extractivists engaged in activities other than pink pepper extraction, with fishing being the most commonly mentioned (22.64%). Most had between two and five years of experience in the activity (35.85%), and 67.92% said they had already faced risks in extracting aroeira.

Regarding land ownership, 66.04% used third-party areas, with 81.13% stating that they operate in freely accessible areas (Table 4). Some extractivists reported that any restrictions on collection in private areas were due to inappropriate exploitation, such as the cutting down of aroeira trees.

Regarding social organization, 50.94% said they participate or have participated in associations, but only 16.98% considered themselves to have a high level of participation. In addition, 98.11% have never received technical assistance. The aroeira collection period, from May to July, was identified as important for income generation, with 39.62% reporting very high profitability and 66.04% perceiving high tree productivity during this period (Table 4).

**Table 4:** Characterization of the productive profile of Pink pepper extractivists. Piaçabuçu, Alagoas, Brazil

Positive indicator	Percentage of respondents %
***Occupation***	
Agriculture	7.55
Extractivism	50.94
Fishing	22.64
Others (local trade, domestic work, general services)	18.87
***Membership in the Association***	
No	49.06
Yes	50.94
***Level of participation in the Association***	
Very low	9.43
Low	1.89
Medium	11.32
High	11.32
Very high	16.98
Does not participate	49.06
***Previous experience with risks***	
No	32.08
Yes	67.92
***Time of extractive experience***	
Less than 2 years	11.32
Between 2 and 5 years	35.85
Between 5 and 10 years	26.42
Between 10 and 15 years	9.43
More than 15 years	16.98
***Property regime***	
Private/third parties	1.89
Public	5.66
Public/third parties	3.77
Third parties	66.04
Third parties/conservation units	7.55
Conservation units	15.09
***Accessibility to collection areas***	
Restricted use	13.21
Restricted use/open access	1.89
Common use	3.77
Open access	81.13
***Profitability***	
Very low	3.77
Low	18.87
Medium	24.53
High	13.21
Very high	39.62
***Productivity***	
Very Low	3.77
Low	1.89
Medium	15.09
High	13.21
Very High	66.04
***Access to Technical Assistance and Rural Extension (ATER) Services***	
No	98.11
Yes	1.89

### The perception of risks and adaptive strategies developed by extractive workers

Participants indicated five risks related to environmental changes, two risks related to accidents, and one risk related to human action. The risk with the highest incidence index was excessive rainfall (0.83), followed by natural accidents (0.62), deforestation (0.40), and temperature increase (0.34) (Table 5).

Most of the adaptive strategies mentioned were associated with risks related to excessive rainfall. The strategy of collecting the fruits, spreading them, and leaving them to dry was reported in 36.36% of respondents’ answers. Other strategies, mentioned less frequently, included: selecting the best fruits and changing the sales method (e.g., accelerating sales or lowering prices to avoid spoilage) (9.09%), altering the species or collection area (4.54%), anticipating the harvest or using tools (4.54%), changing activities (4.54%), and adapting the collection process (2.27%). The absence of strategies to cope with the consequences of excessive rainfall was reported in 20.45% of responses, as participants indicated that they do not collect during these periods.

**Table 5:** Frequency of perceived risk citations, average severity and frequency scores, and number of adaptive strategies mentioned by extractivists for Pink pepper (*Schinus terebinthifolia*)

Risk category	Perceived risk	Incidence index (Ii)	Average severity scores	Average frequency scores	Adaptive strategies and citation percentage (cited strategy/total strategies for that risk)	Total adaptive strategies
Environmental Changes	Increase in Distance	0,17	4,00	4,44	Utilize active transportation (walking or cycling) (66.6%), motorized transport (11.1%), and animal transport (22.2%).	3
	Difficulty of Access	0,08	2,75	2,25	Engage with the owner (50%) and raise awareness among people to refrain from cutting down trees (50%).	2
	Excessive Rainfall	0,83	4,13	2,93	Postpone collection (38.6%), dry and store (36.36%), select during collection and change the sales method (9.09%), change the species or collection area (4.54%), anticipate collection or use a tool (4.54%), change activity (4.54%), and adapt the collection (2.27%).	7
	Increase in Temperature (perceived unusually hot days)	0,34	3,16	2,66	Adapt the collection (36.36%), postpone collection (27.3%), change activity (18.2%), dry and store (9.09%), and select during collection and change the sales method (9.09%).	5
	Deforestation	0,40	4,40	3,84	Adapt the collection (20.8%), anticipate collection or use a tool (20.8%), change the species or collection area (45.8%), and report or engage with people (12.5%).	4
Accidents	Nature-related accidents	0,62	4,00	3,65	Adapt the collection (avoiding or temporarily driving animals away) (42.42%), postpone collection (6.1%), change the species or collection area (39.39%), and anticipate collection or use tools (12.12%).	4
Non-natural	Decline in Value	0,04	5,00	5,00	Postpone collection (50%) and select during collection and change the sales method (50%).	2

### Influence of socioeconomic and productive attributes on the number of perceived risks and adaptive strategies

The final generalized linear models indicated that socioeconomic attributes showed limited explanatory power regarding both the number of perceived risks and the number of adaptive strategies (Table 6). Gender was retained in the model for perceived risks, however, it did not demonstrate a meaningful association with the outcome.

Regarding productive attributes, time of experience in extractivism and profitability exhibited positive tendencies in relation to the number of perceived risks, suggesting that more experienced or economically better-positioned extractivists may perceive a greater variety of risks. Nevertheless, these tendencies did not reach statistical significance and should therefore be interpreted with caution. Productivity, in turn, showed a negative tendency, also without statistical relevance, indicating no consistent relationship with the number of perceived risks.

A similar pattern was observed for the number of adaptive strategies. Among socioeconomic predictors, only the number of information sources remained in the final model, but without demonstrating a relevant effect. Likewise, within the productive attributes, profitability was retained but did not show a statistically meaningful influence on the number of adaptive strategies adopted.

**Table 6:** Final generalized linear models (GLM, Poisson distribution) evaluating the influence of socioeconomic and productive predictors on the number of perceived risks and adaptive strategies among Pink pepper extractivists

Predictor group	Response variable	Predictor (final model)	Estimate Std.	Error	z value	Pr (>|z|)
Socioeconomic	Number of perceived risks	Gender	0.01894	0.13839	0.137	0.8911
	Number of adaptive strategies	Number of sources of information (fon)	−0.1008	0.1117	−0.902	0.367
Productive	Number of perceived risks	Time of experience in extractivism (tex)	0.11578	0.06567	1.763	0.0779
		Profitability (ren)	0.13781	0.07580	1.818	0.0690
		Productivity (pro)	−0.13985	0.09094	−1.538	0.1241
	Number of adaptive strategies	Profitability (ren)	0.08076	0.06730	1.200	0.2301

Only predictors retained after stepwise selection based on the Akaike Information Criterion (AIC) are presented. Estimates correspond to model coefficients; Std. Error = standard error; z value and p-value indicate statistical significance

### Influence of socioeconomic and productive attributes on perceived risk severity

The cumulative link models indicated that socioeconomic attributes showed limited explanatory power regarding perceived risk severity, with age emerging as the only relevant predictor retained in the final model (Table 7). The negative direction of the effect suggests that older extractivists tend to attribute lower severity to the most frequently reported environmental risk, indicating a possible attenuation of perceived threat with increasing age. It is important to note that, in the studied context, age was not directly related to time of experience in extractivism, as might be theoretically expected, since younger adults may sometimes accumulate longer practical experience than older individuals.

Regarding productive attributes, time of experience in extractivism was positively associated with perceived risk severity, suggesting that individuals with longer practical engagement in extractivist activities tend to assign higher severity scores to environmental risks. In contrast, the land tenure regime of the collection area did not demonstrate a consistent or statistically meaningful influence, as the different categories showed no clear pattern of association.

Overall, the ordinal models suggest that perceived risk severity is more strongly influenced by individual experiential factors, such as age and time of engagement in extractivism, than by structural productive conditions related to land access or ownership.

**Table 7:** Final cumulative link model (CLM) evaluating the influence of socioeconomic and productive predictors on perceived risk severity among Pink pepper extractivists

Predictor group	Response variable	Predictor (final model)	Estimate Std.	Error	z value	Pr (>|z|)
Socioeconomic	Perceived risk severity	Age	−0.4806	0.2306	−2.084	0.0371
Productive	Perceived risk severity	Time of extractive experience (tex)	0,628	0,2702	2,325	0,0201
		Public (regb)	−1,5554	2,0213	−0,77	0,4416
		Public/third parties (regb_c)	−0,544	2,0707	−0,263	0,7928
		Third parties (Regc)	1,1314	1,7424	0,649	0,5161
		Third parties/conservation units (regc_d)	3,2974	2,1272	1,55	0,1211
		Conservation units (regd)	3,2159	2,0399	1,577	0,1149

Only predictors retained after stepwise selection based on the Akaike Information Criterion (AIC) are presented. Estimates correspond to ordinal regression coefficients; Std. Error = standard error; z value and p-value indicate statistical significance

## Discussion

### Socioeconomic and productive characteristics of extractivists: evidence of structural inequalities and their consequences for vulnerability

The socioeconomic and productive profiles of pink pepper extractivists reflect a context commonly observed in sociobiodiversity value chains in northeastern Brazil, characterized by the predominance of women, low education, and limited income, which also indicates that this activity may function as an important alternative source of livelihood for individuals with these characteristics. This structural scenario reflects socioeconomic and gender vulnerability, where women play a central role in collection and processing stages but often face restrictions or limited access to resources and markets [52–54].

Vulnerability is further exacerbated by low access to technical and institutional information, with over 98% reporting no technical assistance and only 1.8% accessing more than three sources of information, which may limit opportunities for the development and adoption of innovative adaptive strategies, particularly when new environmental challenges emerge, although horizontal knowledge exchange among extractivists also plays a relevant role in adaptive processes in these production chains [21, 42, 55]. Additionally, the lack of formal guarantees for medium- and long-term resource access, evidenced by the predominance of harvesting on third-party lands (66%) and open-access areas (81%), reflects a resource-use system based on informal and common-use relations. While this increases accessibility under current conditions, it may also weakens community governance and sustainable management, as insecure access reduces decision-making power and long-term incentives for resource stewardship and restoration investments [56].

We observed occupation diversification, which can be interpreted as a strategy to reduce exposure to economic risks, a pattern commonly reported among collectors and rural populations, which may also function as a strategy to reduce exposure to economic risks in a context of environmental change and limited income-generating alternatives. However, the notable economic importance of pink pepper during the harvest season (May to July) renders extractivist income highly seasonal and vulnerable to atypical climatic events. This vulnerability may be further compounded by the disconnect between high field productivity (66%) and low reported profitability (18%), suggesting that the local organizational environment and market relationships need to be strengthened.

This structural context is essential to explain the patterns observed in risk perceptions and adaptive strategies among extractivists in the pink pepper value chain. As a social construct, risk perception may be influenced by structural inequalities, access to information, and integration into the production process.

### Risk perception and adaptive strategies

The risks most frequently cited by extractivists reveal a perception strongly linked to environmental and climatic changes, with particular emphasis on excessive rainfall, deforestation, and rising temperatures. This perception pattern aligns with studies conducted in other extractivist and agro-extractivist systems, which identify environmental changes and the intensification of extreme climatic events as major threats to forest-based production systems [14, 57, 58].

The fact that excessive rainfall represents both the most frequent and highly severe risk highlights the importance of precipitation patterns for the phenological cycle of the pink pepper tree and for the stages of fruit collection and drying. However, the adaptive strategies employed in response to this risk are largely short-term, limited in diversity, and minimally innovative. Although some climatic constraints may be inherently difficult to overcome, this limitation may be further exacerbated by the lack of technical assistance and institutional support networks, which could otherwise expand the range of adaptive responses. Notably, a considerable portion of extractivists reported simply not collecting during periods of excessive rainfall, which may represent a rational short-term response to unfavorable conditions, while also indicating economic vulnerability and limited adaptive options within the pink pepper value chain, as some individuals temporarily shift to alternative activities such as shrimp fishing in flooded areas. While this behavior serves as a functional self-protection mechanism, it results in income loss and further exacerbates economic vulnerability.

### Determinant factors of risk Perceptions, adaptive Strategies, and severity attributed to risks

Our findings revealed that socioeconomic and productive variables did not have a significant effect on the number of perceived risks or the number of adaptive strategies, leading us to reject Hypotheses 1 and 3. However, regarding the severity attributed to risks, the socioeconomic variable age and the productive variable experience in extractivism emerged as significant determinants, supporting Hypotheses 2 and 4.

The relationship between age and perception of environmental change risks presents complex patterns, and evidence points to different scenarios. Large-scale studies, consistent with our findings, indicate that younger individuals tend to perceive climate risks more acutely. For instance, in the study by Paniello-Castillo et al. [28], involving 12,476 individuals in Italy and Sweden, younger participants reported higher perceptions of climate change risks. Similarly, Poortinga et al. [29] found higher levels of climate-related beliefs, risk perceptions, and emotions among younger generations [59].

However, other studies provide contradictory evidence. For example, Gray et al. [30] did not find a correlation between age and perceived severity of environmental losses in their study in the United States. Likewise, Lin et al. [31] observed no significant influence of age on ecological risk perceptions in a study with 243 participants. These divergent findings suggest that environmental, structural, and sociocultural characteristics, as well as methodological choices, may explain differences across studies.

Our results indicate that the severity attributed to risk decreases with age, suggesting that younger and more actively productive extractivists perceive greater severity in environmental change risks within the pink pepper value chain. This perception may be influenced both by more recent and direct exposure to environmental changes and atypical climatic events, as well as by greater exposure to narratives and debates on environmental changes, increasingly disseminated through media over recent decades [28, 59–61].

Recent studies show that exposure to environmental and climate change topics—whether through media or educational processes—is a key factor in understanding generational differences in risk perception. The dissemination of scientific and environmental information over the past decades has helped shape attitudes and increase the sensitivity of younger groups to climatic threats [61, 62]. Consequently, there is a tendency for younger individuals, who grew up with greater access to news, educational campaigns, and public debates on environmental changes, to internalize environmental and climate crisis perceptions more consistently [63]. This helps explain why younger and more informed individuals perceive higher severity regarding environmental risks. In contrast, older members of traditional communities may interpret extreme climatic events, such as excessive rainfall, through an ecological memory of already known natural cycles.

In the studied context, experience in extractivism played a significant role, showing a positive and significant association with the severity attributed to risks. This finding indicates that practical experience enhances real risk perception. More experienced extractivists tend to better recognize environmental change signals and, consequently, are more likely to anticipate potential impacts on resources. Daily interaction with the environment over long periods builds a biocultural memory [37, 64, 65]. However, this process is neither linear nor continuous. In older age, extreme or atypical events may be minimized or normalized, reducing perceived risk, as suggested in studies of traditional systems. For example, Spence et al. (2011) observed that individuals with long histories of exposure to extreme climatic events, such as floods and droughts, tend to incorporate these occurrences into the natural variability of the environment, thereby lowering their perception of threat [29].

It is also important to note that greater experience does not necessarily correspond to older age. In the studied context, pink pepper extractivism strengthened from 2011 onward, with the foundation of the Associação Aroeira. Most extractivists are older than 31 years, which indicates that even younger adults have accumulated a considerable amount of experience in the activity. Moreover, the apparent contrast between the negative effect of age and the positive effect of extractivism experience can be explained by the recent historical consolidation of this value chain in the region. Pink pepper collection is a relatively recent economic activity, intensifying only after the emergence of stable market demand and the formal organization of local associations. Consequently, older individuals are not necessarily those with the longest experience in extractivism, as many began collecting at later stages of life, possibly with lower physical engagement or economic motivation. In contrast, younger or middle-aged extractivists may have accumulated longer and more continuous practical experience, leading to greater familiarity with environmental variability and, therefore, higher perceived severity of risks. This dissociation between chronological age and activity-specific experience reflects a characteristic feature of this socioecological system, in which market demand, rather than traditional subsistence practices, has been the primary driver of participation in pink pepper extractivism.

The significant effect of extractivism experience on the severity attributed to environmental risks observed in our study underscores the importance of practical engagement in shaping threat perceptions in dynamic socioecological contexts. This finding is expected, as continuous interaction with the environment helps individuals learn to recognize early signs of ecological disturbances, especially when their livelihoods depend directly on natural resources. Therefore, while accumulated experience plays a central role in adaptive processes in response to environmental changes [35, 66], recent experiences or memories may exert a strong influence on risk perceptions related to these changes.

Extractivists with longer experience develop a “specialized ecological perception,” defined as the ability to recognize subtle environmental cues and anticipate the severity of risks before they fully manifest [67]. This concept was originally described as empirical knowledge constructed through continuous interaction with the ecosystem, playing a fundamental role in enhancing threat perception and strengthening response capacity to environmental changes [35]. The results of this study support this perspective, indicating that experience in extractivism acts as a relevant mechanism in both the perception of and adaptation to environmental change risks within the value chain.

Recurring experiences with risk events expand the perception of future threats, particularly in agricultural contexts [34]. While we identified experience in extractivism as a predictor of the severity attributed to risks, the variable “prior experience with risks” did not show significance in our model. This discrepancy may reflect the difference between the continuous nature of extractivist activity and the episodic occurrence of risks in other productive practices. Conversely, the lack of a relationship between formal education and risk perception, also noted in previous studies, reinforces our findings regarding the limited influence of formal education in traditional contexts [34]. Complementarily, evidence suggests that past experiences with climate change are associated with the adoption of adaptive strategies, highlighting the importance of lived experience in shaping perceptions and formulating adaptive strategies, even when adapted to the specificities of each socioecological system [29, 36, 57].

It is also important to note that, in addition to environmental threats, traditional communities frequently face growing socioeconomic challenges resulting from restrictions imposed by environmental agencies and private landowners on access to and use of forest resources. In this study, some extractivists reported that the actions of ICMBio, the managing agency of the Piaçabuçu APA, have acted as an obstacle to the continuation of traditional extractivist practices. According to these accounts, the imposition of strict limitations, combined with a lack of effective dialogue, contributes to deepening the economic insecurity of local communities, which rely directly on extractivism as a source of livelihood.

Extractivists’ accounts indicate that restrictions imposed by the managing agency of the Conservation Unit increase community vulnerability. This scenario contrasts with studies recognizing the potential of sustainable use coupled with participatory governance to reconcile local development and conservation [68, 69]. Excluding communities from decision-making processes results in policies that may compromise both social and environmental objectives of protected areas [70]. Thus, institutional misalignment can represent an additional risk, compounding climatic threats and limiting adaptive strategies based on local knowledge.

## Conclusion

Perceptions of environmental change risks are strongly anchored in the practical experience and daily interactions of extractivists with their environment. Age and experience in extractivism are key factors shaping perceptions of the severity of environmental change risks, particularly excessive rainfall, identified as the main risk by the extractivists. In this sense, age highlights generational differences in interpreting risk, while experience in extractivism demonstrates the importance of traditional ecological knowledge in recognizing and responding to environmental changes.

In the studied context, the socioeconomic impacts predominantly affect women over 50 years old, with little or no formal education, earning up to two minimum wages, and having limited access to information sources. In this scenario, extractivism plays a fundamental role not only as a source of incomebut also as a structuring element of autonomy, identity, and territorial permanence for these women.

Given this context, the development of integrated public policies is urgent—policies capable of articulating traditional knowledge, climate justice, and the valorization of extractivist practices. Examples include targeted rural extension programs focused on post-harvest management during periods of excessive rainfall, support for local fruit processing and price-stabilization agreements with cooperatives, and the dissemination of simplified climate forecasts through community communication channels. The construction of adaptive strategies must begin with dialogue with local communities, such as seasonal planning meetings between extractivist associations and environmental agencies and participatory forums for defining access and management rules, recognizing their sociocultural diversity and strengthening their resilience to environmental change impacts. Achieving this requires institutional and political commitment to approaches that promote equity, territorial protection, and the empowerment of traditional populations. Although the patterns identified offer relevant insights into the dynamics of risk perception, it is important to acknowledge that such perceptions may also involve idiosyncratic and context-specific elements that are not fully captured by socioeconomic variables alone. The scarcity of specific and effective strategies to face extreme climatic events raises concerns that extractivists are not adequately prepared to confront future environmental change scenarios.

## Data Availability

Data used to support the findings of this study are available from the authors.

## References

[1] Intergovernmental Panel on Climate Change (IPCC). Climate change 2022: impacts, adaptation and vulnerability. Summary for policymakers. Geneva: IPCC; 2022.

[2] Marques C, Santos F. Riscos e adaptação às mudanças ambientais: os casos de Santos e Ilha Comprida (SP). Ideias. 2021;12:1–27. 10.20396/ideias.v12i00.8665128.

[3] Etkin NL. Edible medicines: an ethnopharmacology of food. Tucson: University of Arizona Press; 2007.

[4] Lujala P. Climate change, natural disasters, and displacements. In: Piguet E, Pecoud A, Guchteneire PD, editors. People on the move in a changing climate. Dordrecht: Springer; 2015. pp. 13–27.

[5] McDaniels TL, Axelrod LJ, Slovic P. Characterizing perception of ecological risk. Risk Anal. 1996;16(5):575–86. 10.1111/j.1539-6924.1995.tb00754.x 7501876

[6] Smith K. Environmental hazards: assessing risk and reducing disaster. 6th ed. London: Routledge; 2017.

[7] Pidgeon N, Fischhoff B. The role of social and decision sciences in communicating uncertain climate risks. Nat Clim Change. 2011;1:35–41.

[8] Weber EU. What shapes perceptions of climate change? Wiley Interdiscip Rev Clim Change. 2010;1(3):332–42. 10.1002/wcc.41.

[9] Birkholz S, Muro M, Jeffrey P, Smith HM. Rethinking the relationship between flood risk perception and flood management. Sci Total Environ. 2014;478:12–20. 24530580 10.1016/j.scitotenv.2014.01.061

[10] González-Riancho C, López-Gutiérrez JS, García-Aguilar O, Gutiérrez OQ, Medina R, Juanes JA. Enhancing resilience of coastal communities to disasters through local perception of risk and knowledge: a case study of the municipality of San Mateo Del Mar, Mexico. Int J Disaster Risk Reduct. 2015;13:47–58.

[11] Shahzad MF, Duan Y, Guo Y. Analysis of climate change perception and adaptation strategies for rice cultivation in Pakistan. Environ Sci Pollut Res Int. 2019;26(12):11691–707.

[12] Sjöberg L. Factors in risk perception. Risk Anal. 2000;20(1):1–11. 10.1111/0272-4332.00001. 10841699

[13] Sjöberg L. The methodology of risk perception research. Qual Quant. 2000;34(4):407–18. 10.1023/A:1004838806793.

[14] Boillat S, Berkes F. Perception and interpretation of climate change among Quechua farmers of bolivia: Indigenous knowledge as a resource for adaptive capacity. Ecol Soc. 2013;18(4):21. 10.5751/ES-05894-180421.

[15] Smit B, Wandel J. Adaptation, adaptive capacity and vulnerability. Glob Environ Chang. 2006;16(3):282–92.

[16] Smith JB, Klein RJT, Huq S. Climate change, adaptive capacity and development. London: Imperial College Press; 2000.

[17] Granderson AA. Making sense of climate change risks and responses at the community level: a cultural-political lens. Clim Risk Manag. 2014;3:55–64.

[18] Rufat S, Tate E, Burton CG, Maroof AS. Social vulnerability to floods: review of case studies and implications for measurement. Int J Disaster Risk Reduct. 2020;47:101547.

[19] Sjöberg L. Risk perception: experts and the public. Eur Psychol. 2000;6(4):226–36.

[20] Coles R, Scott D. Vulnerability and adaptation to climate change and variability in semi-arid rural Southern Africa. Reg Environ Change. 2009;9(3):193–205.

[21] Habtemariam LT, Gari SR, Madariaga M, Leal Filho W. Smallholder farmers’ adaptation to climate variability and change in ethiopia: a multivariate probit assessment. Clim Dev. 2016;8(2):123–32.

[22] Belcher B, Schreckenberg K. Commercialisation of non-timber forest products: a reality check. Dev Policy Rev. 2007;25(3):355–77. 10.1111/j.1467-7679.2007.00374.x.

[23] Shackleton S, Shackleton C, Shanley P, editors. Non-timber forest products in the global context. Berlin: Springer; 2011. 10.1007/978-3-642-17983-9.

[24] Balzon DR, da Silva JCGL, dos Santos AJ. Aspectos mercadológicos de produtos Florestais não madeireiros: análise retrospectiva. Floresta. 2004;34(3):321–30. 10.5380/rf.v34i3.2422.

[25] Mohamed-Katerere JC, Smith M. The role of ecosystems in climate change adaptation: lessons from the Southern African millennium ecosystem assessment (SAfMA). Environ Sci Policy. 2007;10(7–8):648–62.

[26] Davidson DJ, Freudenburg WR. Gender and environmental risk concerns: a review and analysis of available research. Environ Behav. 1996;28(3):302–39. 10.1177/0013916596283003.

[27] McCright AM. The effects of gender on climate change knowledge and concern in the American public. Popul Environ. 2010;32(1):66–87. 10.1007/s11111-010-0113-1.

[28] Paniello-Castillo B, Döring S, Dryhurst S, et al. Risk perception of climate change and global crises: influences of socio-economic drivers and political orientations. Humanit Soc Sci Commun. 2025;12:967. 10.1057/s41599-025-05349-y.

[29] Spence A, Poortinga W, Butler C, Pidgeon NF. Perceptions of climate change and willingness to save energy related to flood experience. Nat Clim Change. 2011;1:46–9. 10.1038/nclimate1059.

[30] Gray E, Raimi KT, Wilson AE, Árvai J. Age differences in climate change beliefs, risk perceptions, and political orientation: a cross-national analysis. Clim Change. 2019;155(1):3–17. 10.1007/s10584-019-02464-9.

[31] Lin J, Zhao X, Zhang B, Li S. The moderating role of age and education in public climate change risk perception: evidence from China. Environ Sci Policy. 2024;159:115–26. 10.1016/j.envsci.2024.03.012.

[32] Kellstedt PM, Zahran S, Vedlitz A. Personal efficacy, the information environment, and attitudes toward global warming and climate change in the united States. Risk Anal. 2008;28(1):113–26. 10.1111/j.1539-6924.2008.01010.x. 18304110 10.1111/j.1539-6924.2008.01010.x

[33] Shi J, Visschers VHM, Siegrist M. Public perception of climate change: the importance of knowledge and cultural worldviews. Risk Anal. 2015;35(12):2183–201. 10.1111/risa.12406. 26033253 10.1111/risa.12406

[34] Magalhães AM, Ferreira GRD, Medeiros PM. Socio-ecological resilience and traditional ecological knowledge: an ethnobotanical study in northeastern Brazil. J Ethnobiol Ethnomed. 2021;17:8. 10.1186/s13002-021-00431-3.33546708

[35] Berkes F, Colding J, Folke C. Rediscovery of traditional ecological knowledge as adaptive management. Ecol Appl. 2000;10(5):1251–62. 10.1890/1051-0761(2000)010[1251:ROTEKA]2.0.CO;2.

[36] Byg A, Salick J. Local perspectives on a global phenomenon—climate change perceptions in a Himalayan community. Glob Environ Change. 2009;19(2):156–66. 10.1016/j.gloenvcha.2009.01.010.

[37] Fernández-Llamazares Á, García RA, Díaz-Reviriego I, Cabeza M, Pyhälä A, Reyes-García V. An empirically tested overlap between Indigenous and scientific knowledge of a changing climate in Bolivian Amazonia. Reg Environ Change. 2015;15(7):1673–83. 10.1007/s10113-014-0725-9.

[38] Adger WN. Social capital, collective action, and adaptation to climate change. Econ Geogr. 2003;79(4):387–404. 10.1111/j.1944-8287.2003.tb00220.x.

[39] Eakin H, Bojórquez-Tapia LA. Insights into the composition of household vulnerability from multicriteria decision analysis. Glob Environ Change. 2008;18(1):112–27. 10.1016/j.gloenvcha.2007.09.001.

[40] Sunderlin WD, Larson AM, Cronkleton P. Forest tenure rights and REDD+: from inertia to policy solutions. In: Angelsen A, editor. Realising REDD+: National strategy and policy options. Bogor: Center for International Forestry Research (CIFOR); 2009. pp. 139–49.

[41] Larson AM, Soto F. Decentralization of natural resource governance regimes. Annu Rev Environ Resour. 2008;33:213–39. 10.1146/annurev.environ.33.020607.095522.

[42] Below TB, Mutabazi KD, Kirschke D, Franke C, Sieber S, Siebert R, Tscherning K. Can farmers’ adaptation to climate change be explained by socio-economic household-level variables? Glob Environ Change. 2012;22(1):223–35. 10.1016/j.gloenvcha.2011.11.012.

[43] Geological Survey of Brazil (CPRM). In: Mascarenhas JC, Beltrão BA, Souza Junior LC, editors. Project for the registration of groundwater supply sources: diagnosis of the municipality of Piaçabuçu, state of Alagoas. Recife: CPRM/PRODEEM; 2005.

[44] Alagoas. State secretariat for planning and economic Development. *Municipal profile: Piaçabuçu*. 3rd ed. Maceió: Government of Alagoas; 2018. p. 35.

[45] Brazilian Institute of Geography and Statistics (IBGE). Piaçabuçu – AL: cities and states. 2022 [cited 2024 May 25]. Available from: https://www.ibge.gov.br/cidades-e-estados/al/piacabucu.html

[46] Verdejo ME. Participatory rural appraisal [Diagnóstico rural participativo]. Brasília: Ministry of Agrarian Development, Secretariat of Family Farming; 2006.

[47] Franco FS. *Diagnosis and design of agroforestry systems in micro-watersheds in the municipality of Araponga, Zona da Mata of Minas Gerais.* Forest Engineering dissertation. Viçosa: Federal University of Viçosa (UFV); 1995. 121 p.

[48] Albuquerque UP, Lucena RFP, Neto EMFL. Selection of research participants. In: Albuquerque UP, Lucena RFP, Cunha LVFC, editors. Methods and techniques in ethnobiology and ethnoecology. 1st ed. New York: Springer Protocols Handbooks; 2014. pp. 1–14.

[49] Baldin N, Munhoz EMB. Snowball: a methodological technique for research in community environmental education. In: National Education Congress; 2011; Curitiba, Brazil. Proceedings. Curitiba: PUCPR; 2011.

[50] Magalhães HF, Oliveira RCS, Feitosa IS, Albuquerque UP. Collection and analysis of environmental risk perception data. In: Albuquerque UP, Lucena RFP, da Cruz Cunha LVF, Alves RRN, editors. Methods and techniques in ethnobiology and ethnoecology. New York: Springer; 2019. p. 149–59. 10.1007/978-1-4614-8636-7.

[51] R Core Team. R: a language and environment for statistical computing. Vienna: R Foundation for Statistical Computing. 2023. Available from: https://www.R-project.org/

[52] Schmitz H, Mota DM, Júnior JF, Jesus NB. Conflitos sociais Em debate: o Caso Das Catadoras de Mangaba no Nordeste e Norte do Brasil. Estud Sociol. 2010;1:157–77.

[53] Souza MHSL, Monteiro CA, Figueredo PMS, Nascimento FR, Guerra RNM. Ethnopharmacological use of Babassu (*Orbignya phalerata* Mart) in communities of Babassu nut breakers in Maranhão, Brazil. J Ethnopharmacol. 2011;133(1):1–5. 10.1016/j.jep.2010.08.056. 20832463 10.1016/j.jep.2010.08.056

[54] Silva RRV, Gomes LJ, Albuquerque UP. What are the socioeconomic implications of the value chain of biodiversity products? A case study in Northeastern Brazil. Environ Monit Assess. 2017;189:64. 10.1007/s10661-017-5772-2. 28105565 10.1007/s10661-017-5772-2

[55] Di Falco S, Kohlin G, Yesuf M. Strategies to adapt to climate change and farm productivity in the Nile Basin of Ethiopia. Clim Change Econ. 2012;3(2):1250009. 10.1142/S2010007812500091.

[56] Larson AM, Cronkleton P, Barry D, Pacheco P. *Tenure rights and beyond: Community access to forest resources in Latin America*. Occasional Paper No. 50. Bogor (Indonesia): Center for International Forestry Research (CIFOR); 2008. 92 p. ISBN 978-979-1412-43-8.

[57] Bauer TN, De Jong W, Ingram V. Perception matters: an Indigenous perspective on climate change and its effects on forest-based livelihoods in the Amazon. Ecol Soc. 2022;27(1):17. 10.5751/ES-12837-270117.

[58] Martínez-Herrera G, Trejo I, Moreno-Calles AI, De Alba-Navarro MF, Martínez-Ballesté A. Knowing the clouds through the land: perceptions of changes in climate through agricultural practices in two Nahua Indigenous communities. J Ethnobiol. 2021;41(3):349–67. 10.2993/0278-0771-41.3.34.

[59] Capstick SB, Whitmarsh L, Poortinga W, Pidgeon NF, Upham P. International trends in public perceptions of climate change over the past quarter century. Wiley Interdiscip Rev Clim Change. 2015;6(1):35–61. 10.1002/wcc.321.

[60] Lee TM, Markowitz EM, Howe PD, Ko C-Y, Leiserowitz AA. Predictors of public climate change awareness and risk perception around the world. Nat Clim Change. 2015;5(11):1014–20. 10.1038/nclimate2728.

[61] van der Linden S. The social-psychological determinants of climate change risk perceptions: towards a comprehensive model. J Environ Psychol. 2015;41:112–24. 10.1016/j.jenvp.2014.11.012.

[62] Stevenson KT, Peterson MN, Bondell HD, Moore SE, Carrier SJ. Overcoming skepticism with education: interacting influences of worldview and climate change knowledge on perceived risk. Environ Educ Res. 2014;20(3):456–71. 10.1080/13504622.2013.812720.

[63] O’Neill S, Nicholson-Cole S. “Fear won’t do it”: promoting positive engagement with climate change through visual and iconic representations. Sci Commun. 2009;30(3):355–79. 10.1177/1075547008329201.

[64] Berkes F, Folke C. Back to the future: ecosystem dynamics and local knowledge. In: Gunderson LH, Holling CS, editors. Panarchy: Understanding transformations in human and natural systems. Washington, DC: Island; 2002. pp. 121–46.

[65] García-del-Amo D, Zant M, Schlingmann A, Reyes-García V. Incremental and transformational adaptation to climate change among Indigenous peoples and local communities: a global review. Mitig Adapt Strateg Glob Change. 2020;25(6):1–25.

[66] Gadgil M, Berkes F, Folke C. Indigenous knowledge for biodiversity conservation. Ambio. 1993;22(2–3):151–6. 10.1007/s13280-020-01478-7 PMC8035379 33566330

[67] Gómez-Baggethun E, Reyes-García V. Reinterpreting change in traditional ecological knowledge. Hum Ecol. 2013;41(4):643–7. 10.1007/s10745-013-9577-1. 10.1007/s10745-013-9577-9 PMC3827740 24244063

[68] Persha L, Agrawal A, Chhatre A. Social and ecological synergy: local rule-making, forest livelihoods, and biodiversity conservation. Science. 2011;331(6024):1606–8. 10.1126/science.1199343. 21436453 10.1126/science.1199343

[69] Ferraro PJ, Hanauer MM. Quantifying causal mechanisms to determine how protected areas affect poverty through changes in ecosystem services and infrastructure. Proc Natl Acad Sci U S A. 2014;111(11):4332–7. 10.1073/pnas.1307712111. 24567397 10.1073/pnas.1307712111 PMC3964111

[70] Ostrom E. Governing the commons: the evolution of institutions for collective action. Cambridge: Cambridge University Press; 2015.

